# Expression of the Myosin Heavy Chain IIB Gene in Porcine Skeletal Muscle: The Role of the CArG-Box Promoter Response Element

**DOI:** 10.1371/journal.pone.0114365

**Published:** 2014-12-03

**Authors:** David M. Brown, John M. Brameld, Tim Parr

**Affiliations:** Division of Nutritional Sciences, School of Biosciences, The University of Nottingham, Leicestershire, United Kingdom; University of Minnesota Medical School, United States of America

## Abstract

Due to its similarity to humans, the pig is increasingly being considered as a good animal model for studying a range of human diseases. Despite their physiological similarities, differential expression of the myosin heavy chain (MyHC) IIB gene (MYH4) exists in the skeletal muscles of these species, which is associated with a different muscle phenotype. The expression of different MyHC isoforms is a critical determinant of the contractile and metabolic characteristics of the muscle fibre. We aimed to elucidate whether a genomic mechanism was responsible for the drastically different expression of MYH4 between pigs and humans, thus improving our understanding of the pig as a model for human skeletal muscle research. We utilized approximately 1 kb of the MYH4 promoter from a domestic pig and a human (which do and do not express MYH4, respectively) to elucidate the role of the promoter sequence in regulating the high expression of MYH4 in porcine skeletal muscle. We identified a 3 bp genomic difference within the proximal CArG and E-box region of the MYH4 promoter of pigs and humans that dictates the differential activity of these promoters during myogenesis. Subtle species-specific genomic differences within the CArG-box region caused differential protein-DNA interactions at this site and is likely accountable for the differential MYH4 promoter activity between pigs and humans. We propose that the genomic differences identified herein explain the differential activity of the MYH4 promoter of pigs and humans, which may contribute to the differential expression patterns displayed in these otherwise physiologically similar mammals. Further, we report that both the pig and human MYH4 promoters can be induced by MyoD over-expression, but the capacity to activate the MYH4 promoter is largely influenced by the 3 bp difference located within the CArG-box region of the proximal MYH4 promoter.

## Introduction

Skeletal muscle is composed of a heterogeneous population of muscle fibres that display a broad spectrum of contractile and metabolic characteristics. The contractile capacity and ATP consumption of a muscle fibre is largely determined by the isotype of the components that constitute the sarcomere [1]. In particular, maximal shortening velocity of a muscle fibre is dictated by the predominant Myosin Heavy Chain (MyHC) isoform expressed [2] and increases in the order of MyHC I<IIA<IIX<IIB. This crucial role of the MyHC isoform to influence the contractile performance of the muscle fibre has resulted in significant interest in understanding the regulation of different MyHC isoforms [3], [4]. Although regulation of the slower (type I and IIA) MyHC isoforms is relatively well understood [5], [6], [7], [8], knowledge of the mechanisms regulating the gene expression of the faster contracting, type IIB MyHC isoform still remains sparse and incomplete [9], [10].

Despite all mammals carrying the gene (MYH4) encoding MyHC IIB, extensive characterization has revealed a striking divide between large and small mammals with regard to MYH4 expression. Most small mammals such as mice [2], rats [2], rabbits [2], [11], and guinea pigs [12], [13] express MYH4 but most larger mammals such as cows [14], sheep [15] horses [16], goats [17], cats [18], [19], dogs [20], baboons [19] and humans [2], [21] do not express. Interestingly, domesticated pigs remain an anomaly amongst the large mammals as they express high levels of MYH4 [22], [23], a phenotype likely exacerbated by the intensive selection pressure for enhanced muscle growth in these animals [24], [25]. This expression of MYH4 in pig skeletal muscle is associated with a dramatically faster and more glycolytic muscle phenotype compared to that of human skeletal muscle. Given the impact of MyHC isoform expression on the contractile and metabolic characteristics of the muscle fibre, it is interesting to explore the mechanisms dictating this unusually high expression of MYH4 in pig skeletal muscle, which currently remains unknown.

Previous studies on the MYH4 promoter have elucidated several key regions of regulatory control (mAT 1/2/3, CArG-box and Ebox) but have predominantly been conducted on the mouse promoter sequence (a MYH4 expressing species) [26], . A recent study [32] extended this work to compare the activity of the human and mouse MYH4 promoter and revealed that a single base pair mismatch in the human CArG-box sequence (−74 bp relative to the TATA-box) results in reduced MYH4 promoter activity, relative to the equivalent mouse promoter.

Differences in the regulation of MYH4 expression between rodent species and humans might be expected as their muscle metabolism, and therefore their contractility characteristics, are different, probably reflecting the metabolic constraints caused by body size [33]. Pigs, on the other hand, are physiologically similar to humans and are increasingly being considered as a good animal model for studying a range of human diseases [34]. It is therefore important to determine what differences exist between these species in order to have confidence in the use of pigs as a model. Given that pigs also exhibit high expression of MYH4, unlike humans and most large animals, we set out to determine whether the mechanisms by which this is achieved is also through variations in promoter sequence, as is the case in mice when compared to humans [32]. We hypothesized that genomic differences in the MYH4 promoter of pigs and humans dictate the differential expression of MYH4, which is associated with a different muscle phenotype in these otherwise physiologically similar mammals. Using an *in vitro* fluorescence based promoter-reporter system; we utilized approximately 1 kb of the MYH4 promoter from a domestic pig and a human to elucidate the role of the promoter sequence in regulating the high expression of MYH4 in porcine skeletal muscle.

## Materials and Methods

### Cloning

Approximately 1 kb of the pig and human MYH4 promoters (−961 bp to +32 bp, relative to the TATA box) was generated by polymerase chain reaction (PCR) and cloned into the pZsGreenI-I fluorescence-reporter plasmid (Clontech) for transfection studies in the C2C12 mouse myoblast cell line. All base pair numbering is relative to the TATA-box, as a discrepancy existed as to the location of the transcription start site between the two promoters. Accession numbers for the pig and human MYH4 gene (NM_001123141.1 and ENSG00000264424.1, respectively) were used to locate the first exon and the 3′ oligonucleotide primer was designed to span into the first exon to ensure inclusion of the transcription start site. All oligonucleotide sequences are displayed in Table 1.

**Table 1:** Oligonucleotide sequences used for cloning. Bold underline indicates base pairs being mutated.

Oligonucleotide name	Sequence (5′-3′)
Human_MYH4_R	AAAAAACCCGGGAGGAAGGATGGGAAAGAGG
Pig_ MYH4_R	AAAAAACCCGGGAGGAAGGACAGGACAGAGGCAT
Human_ MYH4_67bp_F	AAAAAACTCGAGGCCAAGTAGGTTCCCAGCTAG
Pig_ MYH4_67bp_F	AAAAAACTCGAGGCCAAGTAGGTTCCCAGCTGG
Human+pig_ MYH4_113bp_F	AAAAAACTCGAGCCACAGTCAGTGAATATTGTGCA
Human_ MYH4_231bp_F	AAAAAACTCGAGCAGTGTTAGAAGCCCTGAATCC
Pig_ MYH4_231bp_F	AAAAAACTCGAGTAGCCTTAGAAGCCCTGAATCTC
Human_ MYH4_466bp_F	AAAAAACTCGAGTCCAGACTGTGGCTAGGAATG
Pig_ MYH4_466bp_F	AAAAAACTCGAGTCATCAGTATTTCTAAAATCCAGACC
Pig_961bp_(1kb)_ MYH4_F	AAAAAACTCGAGTAGGTGACACACTTAGCGTGGACA
Human_961bp_(1kb)_ MYH4_F	AAAAAACTCGAGCTTAGAGTAGGTATATTTCCTGCCAT
Human_ MYH4_HindIII_F	CAGTGTTAGAAGC**TT**TGAATCCCCATC
Human_ MYH4_HindIII_R	TTTCTCCAGTTTGGCTGTTGCATC
Pig_ MYH4_HindIII_F	GCCTTAGAAGC**TT**TGAATCTCCATC
Pig_ MYH4_HindIII_R	TATTTCTCCAGCTTTGCTGTTCCATC
Human_ MYH4_AT3_SDM_F	CTATCAAATGCCT**CA**AAAGAACCCTAGA
Human_ MYH4_AT3_SDM_R	GGGATGGGGATTCAGGGC
Human_ MYH4_midAT2-3_SDM_F	GAACCCTAGAT**G**ATCCTCTGTTAAAT
Human_ MYH4_midAT2-3_SDM_R	TTTATAGGCATTTGATAGGGGATGG
Human_ MYH4_distAT2_SDM_F	CTAGATCATCCTCTGT**C**AAATTATTTATGGG
Human_ MYH4_distAT2_SDM_R	GGTTCTTTATAGGCATTTGATAGGGG
Human_ MYH4_proxAT2_SDM_F	CCTCTGTTAAATTATTTA**T**AGGTGTCAAGAAATA
Human_ MYH4_proxAT2_SDM_R	ATGATCTAGGGTTCTTTATAGGCATT
Human_ MYH4_CArG_SDM_F	AGATTGCCAAAAA**A**GGTTTTGCCAAG
Human_ MYH4_CArG_SDM_R	CTTTGCACAATATTCACTGACTGTGG
Human_ MYH4_EBOX2_SDM_F	TTCCCAGCT**G**GGACAGCT
Human_ MYH4_EBOX2_SDM_R	CCTACTTGGCAAAACCGTTTTTGGC
Human_ MYH4_T_removal_F	GCCAAGTAGGTTCCCAGCT
Human_ MYH4_T_removal_R	AAACCTTTTTTGGCAATCTCTTTGCAC
No-CArG_R	CTCTTTGCACAATATTCACTGACTGTG
No-CArG_Pig_ MYH4_F	AGTAGGTTCCCAGCTGGG
No-CArG_Human_ MYH4_F	AGTAGGTTCCCAGCTAGGAC
CMV-basic_F	AAAAAAAAGCTTGCAAATGGGCGGTAGGC
CMV-basic_R	AAAAAACCCGGGGATCTGACGGTTCACTAAACCAGC
MyoD_F	AAAAAAAGATCTGCCACCATGGAGCTTCTATCGCCG
MyoD_R	AAAAAAACCGGTGCTCAAAGCACCTGATAAATCGC

Pig and human MYH4 promoters were subjected to 5′ deletion analysis creating promoters of varying lengths (−961 bp, −466 bp, −231 bp, −113 bp, −67 bp; promoter lengths are relative to the TATA-box). Site directed mutagenesis and site-specific deletions within the MYH4 promoters were introduced by PCR (specific base pair substitutions are described in the results section).

Chimeric MYH4 promoters (containing combinations of both distal and proximal regions of the pig and human MYH4 promoters) were produced according to a design previously demonstrated [32]. A HindIII restriction site (2 bp mutation; at −218 bp and −217 bp) was introduced to both the pig and human 1 kb promoter allowing the proximal promoter regions to be swapped by restriction digest, generating chimeric promoters. Proximal promoter regions were also swapped with a minimal CMV promoter (84 bp) to test for potential regulatory regions in the 5′ distal region (∼750 bp) of each promoter, independent of the proximal promoter.

Full-length open reading frame MyoD cDNA (accession number: ENSMUST00000072514.1) was generated by RT-PCR, cloned into the pDsRed-Express-N1 plasmid (clontech) and over-expressed in C2C12 cells under the control of a CMV promoter. A pDsRed-Express-N1 plasmid lacking the CMV promoter was used as a vehicle transfection control.

### Electrophoretic mobility shift assay

Protein-DNA interactions were assessed by incubating C2C12 myotube nuclear extracts with a 5′ biotin labeled double-stranded probe for a 62 bp region spanning (and inclusive of) the CArG-box and Ebox2 sequence (−93 bp to −31 bp relative to the TATA-box) from the pig MYH4 promoter (probe sequences are in Table 2). Three shorter un-labeled probes, from the same region (pig sequence), were used to compete for bound proteins and identify the specific regions within the 62 bp probe interacting with nuclear protein(s). Three equivalent human un-labeled probes were used to assess whether the species-specific sequence variation in these regions impaired competition for the proteins bound to the pig 62 bp labeled probe. Unlabeled competitor probes were designed to the following regions, CArG-box: −91 bp to −60 bp, mid-probe: −74 bp to −51 bp and E-box2 probe: −63 bp to −31 bp, all relative to the pig TATA-box.

**Table 2:** Probe sequences used for electrophoretic mobility shift assays.

EMSA probe name	EMSA probe sequence (5′ to 3′)
Pig_ MYH4_probe_sense (biotin-labelled)	TGCAAAGAGATTGCCAAAAAAGGTTTGCCAAGTAGGTTCCCAGCTGGGACAGCTGAGGTGGC
Pig_ MYH4_probe_anti-sense (biotin-labelled)	GCCACCTCAGCTGTCCCAGCTGGGAACCTACTTGGCAAACCTTTTTTGGCAATCTCTTTGCA
Human_ MYH4_CArG-competitor_sense	AAGAGATTGCCAAAAACGGTTTTGCCAAGT
Human_ MYH4_CArG-competitor_anti-sense	ACTTGGCAAAACCGTTTTTGGCAATCTCTT
Human_ MYH4_mid-probe-competitor_sense	CGGTTTTGCCAAGTAGGTTCCCA
Human_ MYH4_mid-probe-competitor_anti-sense	TGGGAACCTACTTGGCAAAACCG
Human_ MYH4_E-box2-competitor_sense	AGTAGGTTCCCAGCTAGGACAGCTGAGGTGGC
Human_ MYH4_E-box2-competitor_anti-sense	GCCACCTCAGCTGTCCTAGCTGGGAACCTACT
Pig_ MYH4_CArG-competitor_sense	AAGAGATTGCCAAAAAAGGTTTGCCAAGT
Pig_ MYH4_CArG-competitor_anti-sense	ACTTGGCAAACCTTTTTTGGCAATCTCTT
Pig_ MYH4_mid-probe-competitor_sense	AGGTTTGCCAAGTAGGTTCCCA
Pig_ MYH4_mid-probe-competitor_anti-sense	TGGGAACCTACTTGGCAAACCT
Pig_ MYH4_E-box2-competitor_sense	AGTAGGTTCCCAGCTGGGACAGCTGAGGTGGC
Pig_ MYH4_E-box2-competitor_anti-sense	GCCACCTCAGCTGTCCCAGCTGGGAACCTACT

Protein-DNA binding conditions consisted of 50 ng/ul Poly dI-dC, 5% glycerol, 0.05% NP-40, 50 mM KCL, 1 mM MgCl_2_, 1 mM EDTA, 20 fmol biotin-labeled probe, 4 pmol un-labeled competitor probe and 3 µl of C2C12 myotube nuclear extract (from NE-PER extraction kit, Thermo Scientific) in 1x binding buffer (LightShift Chemiluminescent EMSA kit, Thermo Scientific) in a total volume of 20 µl. Protein-DNA binding reactions were incubated at room temperature for 20 minutes, separated by electrophoresis on a 6% non-denaturing polyacrylamide gel (Invitrogen, Novex) at 100 V for 75 minutes and transferred by electro-blotting to a Biodyne-B nylon membrane (Thermo Scientific) at 100 V for 45 minutes. Protein-DNA complexes were cross-linked for 60 seconds (auto-crosslink) and biotin-labeled probes were detected using the LightShift Chemiluminescent EMSA detection module (Thermo Scientific) according to the manufacturer's instructions.

### Cell culture and transfections

C2C12 mouse myoblasts were cultured in proliferation medium (DMEM, 10% fetal bovine serum, 1% penicillin and streptomycin) and induced to differentiate at confluence by switching to differentiation medium (DMEM, 2% horse serum, 1% penicillin and streptomycin) for up to 6 days. Medium was refreshed every 48 hours.

Myoblasts (at ∼70% confluence) were transfected with the pig or human 1 kb MYH4 pZsGreenI-I promoter-reporter plasmid using FugeneHD (Promega) transfection reagent at a ratio of 3∶1 (FugeneHD:DNA). Each well was co-transfected with pDsRed-Express-N1 as a transfection efficiency control for normalizing ZsGreenI-I promoter-reporter fluorescence. MYH4 promoter activities (fluorescence output) were quantified following 5 or 6 days of differentiation using the Typhoon Trio+ (GE healthcare). MyoD over-expression plasmids (or pDsRed-Express-N1 plasmids lacking the CMV promoter) were transfected into myoblasts at the same time as the MYH4 promoter-reporter plasmids at a ratio of 1∶1.

### Statistical analysis

All fluorescence promoter-reporter assays were conducted using 4 biological replicates (wells). Data is displayed as mean ± standard deviation (SD). Statistical analysis was conducted using GenStat (version 15) and significance was accepted at *p*<0.05.

## Results

**Figure 1:**
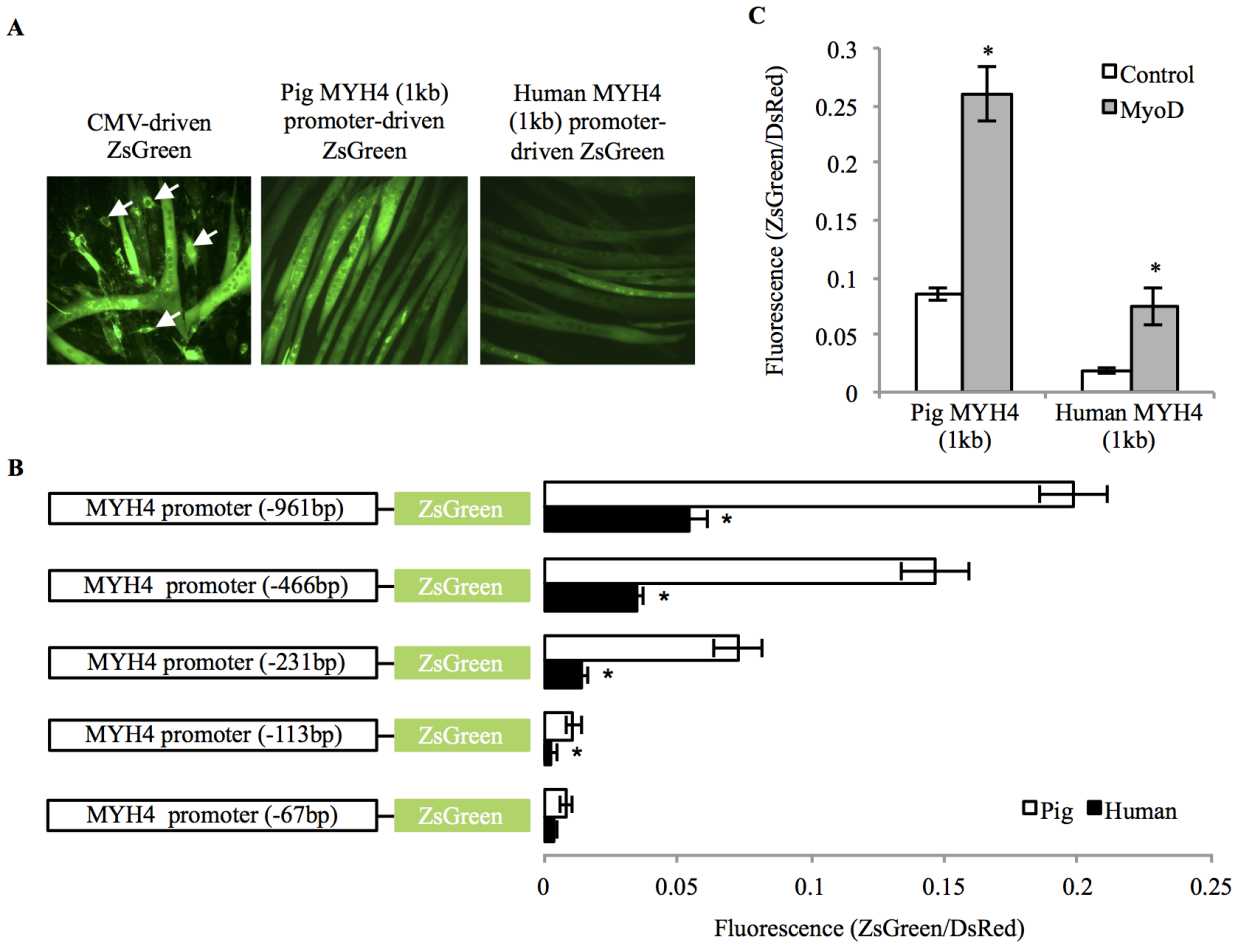
Pig and human MYH4 promoter activities in C2C12 muscle cells. (A) Representative fluorescence images show 1 kb MYH4 promoter-reporter constructs are differentiation specific. (B) Equivalent 5′ deletion analysis of the pig and human MYH4 promoters. Promoter activities in Day 6 differentiated C2C12 myotubes (mean ± SD). * Indicates Human MYH4 promoter activity was significantly different to the equivalent length pig promoter (*p*<0.05). (C) 1 kb pig and human MYH4 promoter activity in response to (mouse) MyoD over expression in day 5 differentiated C2C12 myotubes (mean ± SD). * Indicates MYH4 promoter activity was significantly different to the control transfection (*p*<0.05).

**Figure 2:**
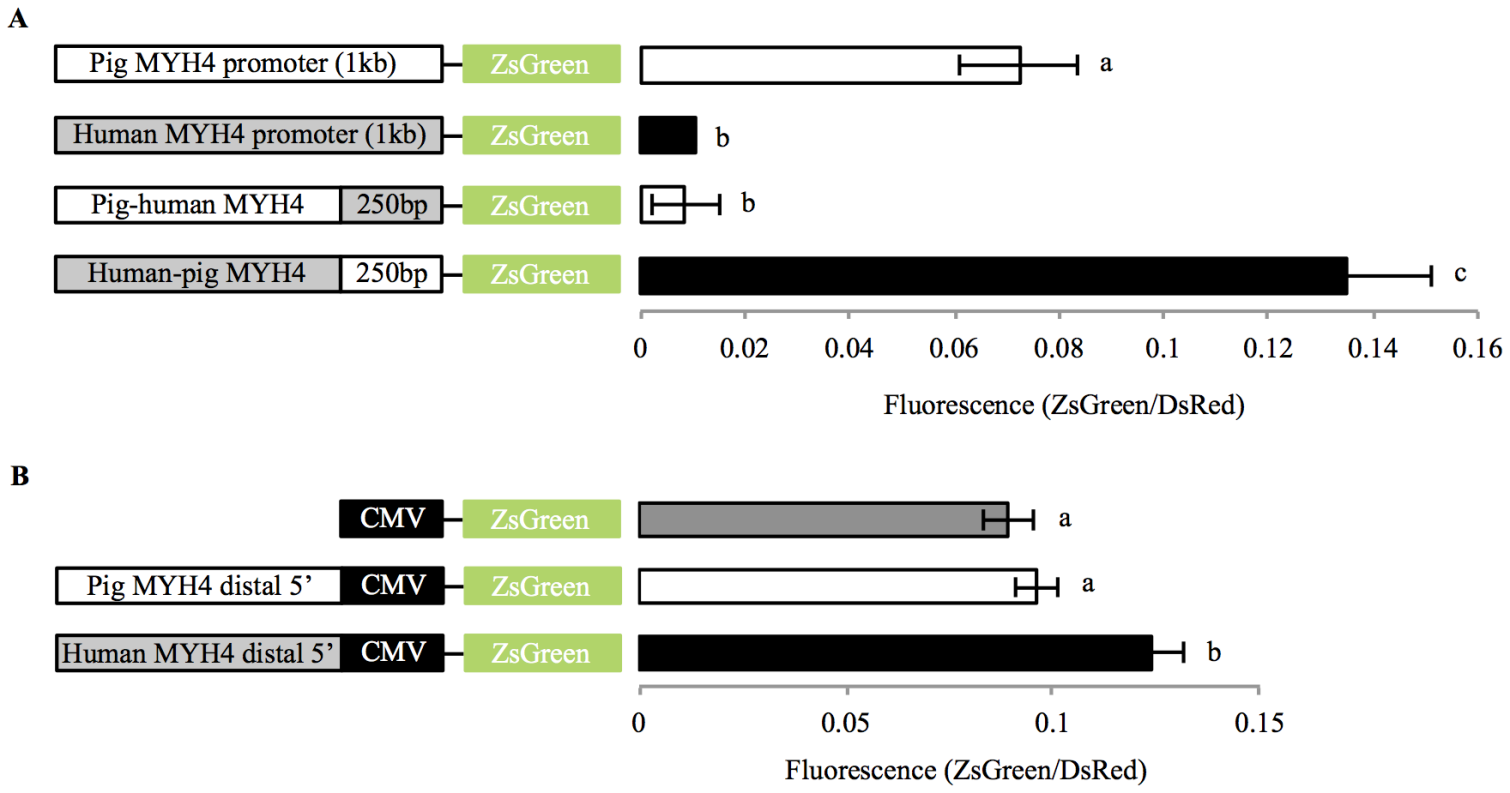
Pig and Human chimeric MYH4 promoter activities in C2C12 muscle cells. (A) Pig-human and human-pig (5′ to 3′) chimeric MYH4 promoters were constructed to assess the role of the proximal 250 bp of each promoter within the context of a 1 kb promoter. (B) The distal (∼750 bp) of the pig and human MYH4 promoters were cloned upstream of a minimal CMV promoter (84 bp) to assess whether this region elicits enhancer activity independent of the proximal (∼250 bp) MYH4 promoter (mean ± SD). Differing letters (a,b,c) constitute a significant difference between promoter activities (*p*<0.05).

Alignment of the pig and human MYH4 promoter sequences revealed very high similarity (1 kb = 80% identical; proximal 200 bp = 95% identical). Following transfection into mouse C2C12 muscle cells, activity of the 1 kb pig and human MYH4 promoters was restricted to differentiated myotubes (indicated by ZsGreen fluorescence), unlike a CMV-driven ZsGreen control, which was expressed in myoblasts and myotubes; see Figure 1A). Despite both promoters being active in differentiated C2C12 myotubes, the 1 kb pig MYH4 promoter had higher activity than the equivalent human promoter, which was only mildly active (Figure 1A and 1B; *p* = 0.006). Due to the disparity in MYH4 promoter induction during myogenesis, we asked whether MyoD, a myogenic regulatory factor known to induce MYH4 expression [10], [29], [30], [32], was activating the pig, but not human MYH4 promoter during C2C12 myogenesis. Over-expression of mouse MyoD during C2C12 differentiation was capable of inducing both the pig and human MYH4 promoters by ∼3 and ∼4-fold respectively (Figure 2A; *p*<0.01). Fluorescence microscopy revealed that MYH4 promoter activity was still restricted to differentiated myotubes in the presence of MyoD over-expression (data not shown). Although both the pig and human MYH4 promoters were induced by a myogenic signal (MyoD), the absolute capacity for induction of the human MYH4 promoter was significantly restricted in comparison to the pig MYH4 promoter activity. It was therefore postulated that response elements within the human MYH4 promoter were limiting the capacity to fully activate the promoter and that the pig MYH4 promoter contained response elements that permitted a much greater potential for activation during myogenic differentiation.

In order to locate regulatory regions controlling the differential activity of the pig and human 1 kb MYH4 promoters, we conducted equivalent 5′ deletion analysis of both promoters. Disparity in pig and human MYH4 promoter activity persisted with decreasing promoter length (from −961 bp to −113 bp) and declined similarly for promoters from both species (Figure 1B). Removal (by 5′ deletion) of ∼120 bp, between −231 bp to −113 bp, essentially removing the AT-rich regions (AT 1, 2 and 3), caused the largest reduction in promoter activity for both the pig and human MYH4 promoters (84% and 82% respectively). Deletions at the 5′ end of the pig and human MYH4 promoters from −961 bp to −466 bp caused a 26% and 37% reduction, respectively, and deletion from −466 bp to −231 bp caused a 50% and 58% reduction, respectively. The largest disparity in promoter activity between the pig and human MYH4 promoters existed in the −231 bp promoters (∼5-fold difference; *p*<0.001), indicating that response elements controlling high and low MYH4 promoter activity likely resided downstream of −231 bp.

**Figure 3:**
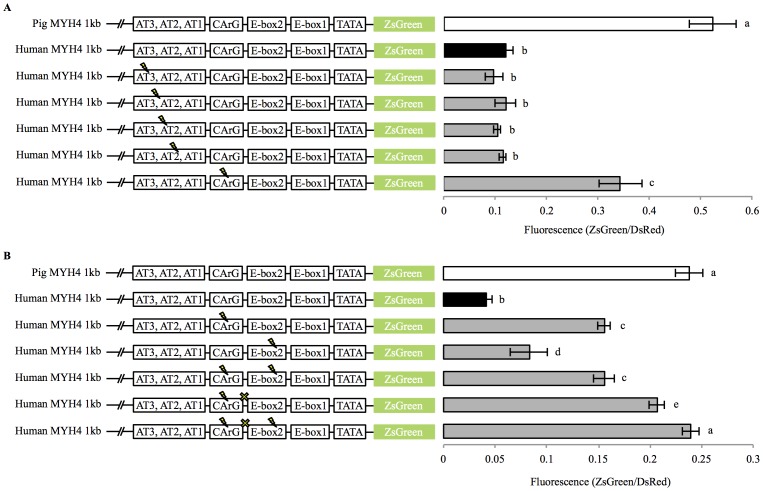
Mutated human MYH4 promoter activity in C2C12 muscle cells. (A) Site directed mutagenesis within the AT-rich region and the CArG-box of the 1 kb human MYH4 promoter (substitutions indicated by a “bolt” symbol). (B) Site directed mutagenesis within the CArG/Ebox2 region of the 1 kb human MYH4 promoter. A “bolt” indicates a single base pair substitution and a “cross” indicates removal of a single base pair. Promoter activities were measured in day 6 differentiated C2C12 myotubes (mean ± SD). Differing letters (a,b,c,d,e) constitute a significant difference between promoter activities (*p*<0.05). Open bar  =  pig promoter; black bar  =  human promoter; grey bar  =  mutant human promoter.

To confirm the role of the proximal promoter sequence in dictating high and low MYH4 promoter activity, we produced chimeric MYH4 promoters (generated by swapping the proximal region (−218 bp to +32 bp) of the pig and human promoters). When the proximal region of the pig 1 kb MYH4 promoter was replaced with the equivalent human proximal promoter, activity was reduced (*p*<0.001) to the same level as the human 1 kb MYH4 promoter (Figure 2A; *p* = 0.627). When the proximal region of the human 1 kb MYH4 promoter was replaced by the equivalent pig proximal promoter, activity was increased (*p*<0.001) and interestingly exhibited an almost 2-fold greater activity than the 1 kb pig MYH4 promoter (*p* = 0.005). The chimeric promoters confirmed that sequence differences in the proximal promoter regions (totaling 14 bp differences) dictate the high and low MYH4 promoter activities. For completeness, the pig and human proximal MYH4 promoters (−218 bp to +32 bp) were replaced by an 84 bp minimal CMV promoter, which revealed that, the 5′ distal human (*p*<0.001), but not pig (*p* = 0.135), MYH4 promoter region was capable of increasing the basal promoter activity of the minimal CMV promoter (Figure 2B). This highlights that the proximal MYH4 promoter region contains the key response elements required to elicit differential promoter activities between pigs and humans. To identify the specific sequence differences responsible for the differential pig and human MYH4 promoter activities, we analyzed the effects of site directed mutagenesis on promoter activities in C2C12 myotubes. Site directed mutagenesis within the AT rich regions (mAT2 and mAT3) of the human 1kb MYH4 promoter (making base pair alterations to match the equivalent pig promoter), had no impact on MYH4 promoter activity (Figure 3A). However, a single base pair alteration within the human CArG-box (at −74 bp), was sufficient to increase promoter activity by ∼2.5-fold (*p* = 0.001), but promoter activity remained lower than the 1 kb pig MYH4 promoter (*p* = 0.007; Figure 3A).

Further site directed mutagenesis downstream of the CArG-box in the human MYH4 promoter highlighted a group of 3 bp mutations required to increase promoter activity to the same level as the pig MYH4 promoter (Figure 3B). A single base pair mutation within the human CArG-box sequence (at −74 bp) increased promoter activity (*p*<0.001) but remained lower than the activity of the 1 kb pig MYH4 promoter (*p*<0.001). Removal of a single nucleotide (at −68 bp) from the human MYH4 CArG-box mutant promoter resulted in a further increase in promoter activity, compared to the CArG-box mutant promoter (*p*<0.001), but this level of activity still remained lower than the 1 kb pig MYH4 promoter (*p* = 0.023). Finally, a combination of the CArG-box mutation (at −74 bp), with the removal of a single nucleotide (at −68 bp), and a single base pair change in the E-box2 (at −48 bp) caused an increase in human MYH4 promoter activity (*p*<0.001) to a level equal to the 1 kb pig MYH4 promoter (*p* = 0.895).

**Figure 4:**
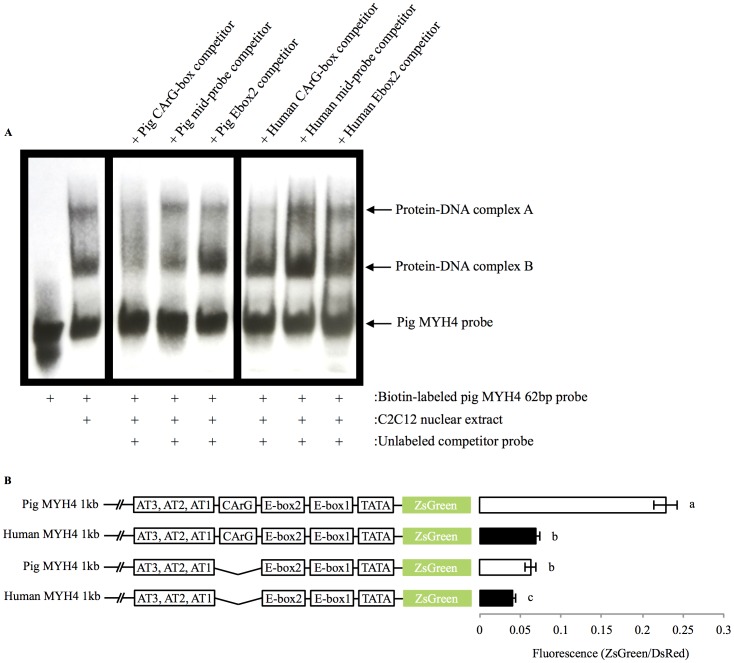
The role of the CArG-box promoter response element in pig and human MYH4 promoter activity. (A) Electrophoretic mobility shift assay using a 62 bp biotin labeled probe (spanning the pig CArG and Ebox2 region; −91 bp to −31 bp relative to the TATA-box). Probes were incubated with C2C12 myotube nuclear extracts. Cross-species competition for bound proteins was conducted using shorter un-labeled pig and human probes. The human CArG-box is unable to bind proteins forming complex B with the pig CArG-box. Experiments were repeated 3 times to confirm the results. (B) Removal of the CArG-box region (total of 22 bp removed) from the 1 kb pig and human MYH4 promoters. Promoter activities were measured in day 6 differentiated C2C12 myotubes (mean ± SD). Differing letters (a,b,c) constitute a significant difference between promoter activities (*p*<0.05).

Electrophoretic mobility shift assays were conducted to determine whether the 3 bp mismatch, responsible for the disparity in promoter activity between the pig and human MYH4 promoters, caused differential nuclear protein binding to this region (Figure 4A). Two protein-DNA complexes (labeled A and B) formed with the pig 62 bp probe (spanning and inclusive of the CArG-box and Ebox2 (−93 bp to −31 bp)). To identify specific regions of the 62 bp probe bound by protein(s), 200-fold molar excess of shorter, un-labeled competitor probes were included in the binding reactions. The un-labeled pig CArG-box probe (29 bp in length) bound the proteins forming both complexes A and B with the 62 bp pig probe, as both bands were lost/reduced (Figure 4A). The un-labeled pig mid-probe and Ebox2 probes, however, were unable to compete for proteins bound to the 62 bp probe, as there were no effects on either band. Interestingly, proteins bound to the pig CArG-box region were sensitive to the species-specific differences at −74 bp and −68 bp in the pig and human CArG-box region (2 bp mismatch). The un-labeled human CArG-box competitor probe was capable of competing with the pig 62 bp probe for proteins forming complex A, but not B. Like the equivalent pig competitor probes, the human mid-probe and Ebox2 probes were also unable to compete for proteins bound to the 62 bp pig probe. Therefore, the 2 bp mismatch at −74 bp and −68 bp in the pig and human CArG-box region resulted in differential protein-DNA interactions, highlighting the functional importance of sequence variation in this genomic region.

Finally, to confirm the role of sequence variation at −74 bp and −68 bp in the CArG-box region to dictate the strength of porcine MYH4 promoter induction, the CArG-box region (−63 bp to −84 bp; inclusive of the two mismatches at −74 bp and −68 bp) was deleted from the pig 1 kb MYH4 promoter, which reduced activity (*p*<0.001; Figure 4B) to the same level as the 1 kb human MYH4 promoter (*p* = 0.134). Despite sequence differences in this region, removal of the equivalent CArG-box region from the 1 kb human MYH4 promoter also moderately reduced promoter activity (*p*<0.001; Figure 4B).

**Figure 5:**
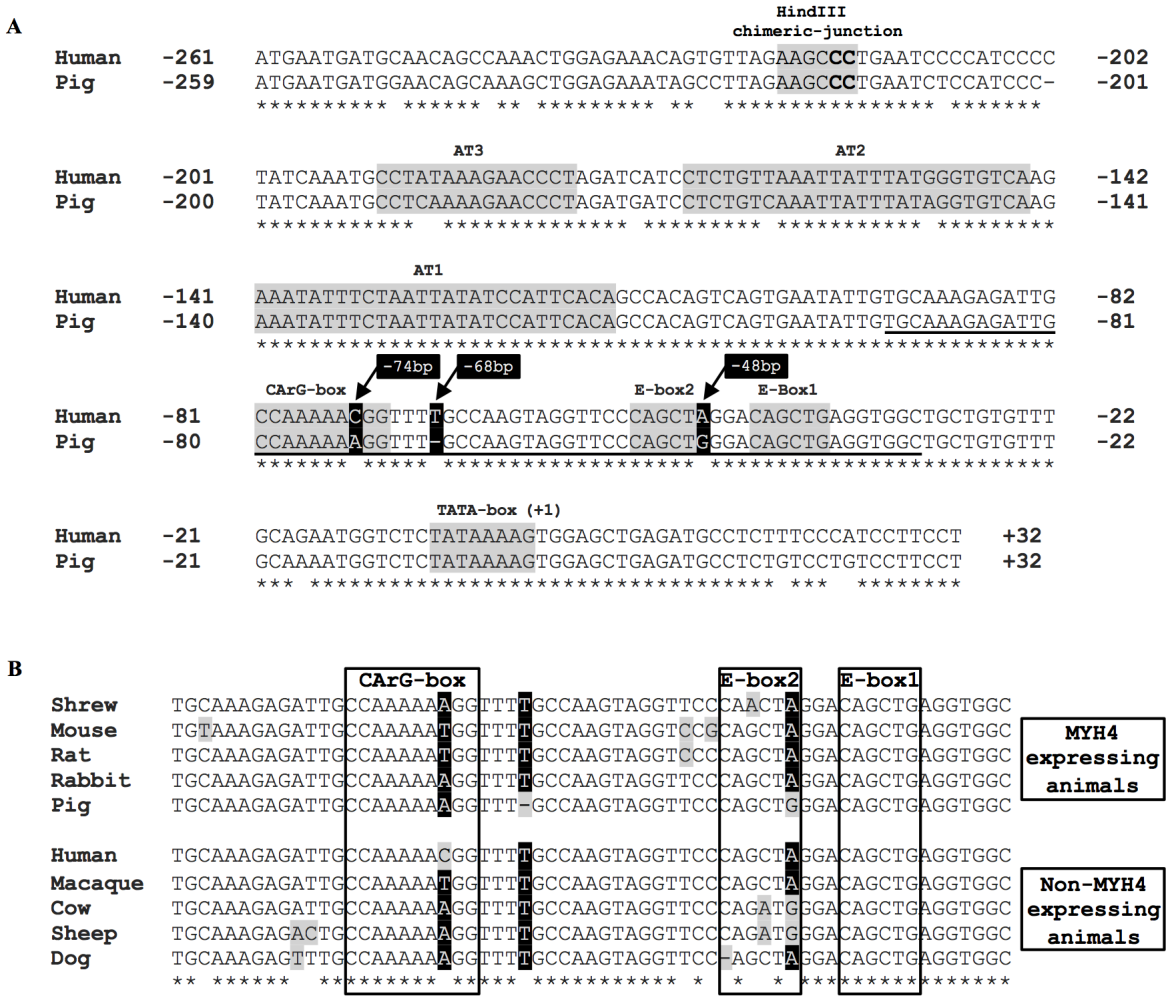
Comparison of the evolutionary conserved CArG-box and E-box region in MYH4 expressing and non-expressing species. (A) Alignment of the proximal pig and human MYH4 promoter. All base pair numbering is relative to the respective TATA-box (+1). Base pair mismatches, highlighted in black (with base pair numbering relative to the human TATA-box), were responsible for the differential activity of the 1 kb pig and human MYH4 promoter in C2C12 myotubes. Bold-underline indicates the 62 bp pig probe used in EMSA experiments (spanning the CArG-box and E-box region (−93 bp to −31 bp)). The two bold base pairs (**CC**) indicate the mutated HindIII site for generating chimeric-promoters. (B) Cross-species alignment of the CArG-box and E-box region from MYH4 expressing and non-expressing animals. Bases highlighted in the black columns indicate the location of crucial base pair differences required for high MYH4 expression in pigs relative to humans. There is no common mutation amongst MYH4 expressing and non-expressing species, but there are a number of differences (highlighted in grey) within this highly conserved region, which may be critical to the strength of promoter activation.

An alignment of the proximal pig and human MYH4 promoter (Figure 5A) highlighted the close proximity of the 3 bp mismatches in the CArG and E-box region that are responsible for the high level activity of the porcine MYH4 promoter. An additional alignment of this CArG and E-box region from multiple MYH4 expressing and non-expressing species (Figure 5B) identified that there is no common variation in sequence that is responsible for the differential expression of MYH4, but sequence variation within this region does exist between species.

## Discussion

Myosin heavy chain (MyHC) isoform expression is a key determinant of the contractile performance of the muscle fibre, with maximal shortening velocity increasing in the order of MyHC I<IIA<IIX<IIB [2]. Mechanisms regulating the expression of the different MyHC isoforms in skeletal muscle have therefore been of significant interest within the scientific community [3], [4]. Interestingly, extensive characterization has revealed a species differential expression of the fastest contracting isoform, MYH4, which is associated with different muscle phenotypes between species. Of particular interest, domestic pigs express very high levels of MYH4 [22], [24], [25], unlike most large mammals (which do not normally express MYH4), and exhibit a faster and more glycolytic muscle phenotype in comparison. We examined the role of the MYH4 promoter sequence in dictating the expression of MYH4 in pigs, compared to humans, which do not express MYH4 [2], [21]. Due to its similarity to humans, the pig is increasingly being considered as an animal model for a range of human diseases [34] and it is therefore useful to understand the mechanisms responsible for the physiological differences between these species. We identify a genomic difference within the MYH4 promoter of pigs and humans that dictate differential promoter activity during myogenesis and postulate that these genomic differences partly explain the different MYH4 expression profiles in these otherwise physiologically similar mammals.

We previously reported that the C2C12 cell line is a dynamic MYH4 expressing environment, with mRNA expression increasing throughout the latter stages of myogenic differentiation [35]. This cell line was selected for transfection studies of the pig and human MYH4 promoter-reporter constructs, providing a common and non-biased MYH4 inducing environment for testing both promoters. In this environment, 1 kb of the pig MYH4 promoter elicited very strong activity that is consistent with the very high level of endogenous MYH4 expression observed in pigs [22], [23]. In contrast, the human 1 kb MYH4 promoter was only mildly active during C2C12 myogenic differentiation. We were slightly surprised by the extent of the human MYH4 promoter activity in C2C12 cells given that humans do not normally express MYH4 transcripts *in vivo* [2], [21], suggesting that this promoter can display mild activity given the appropriate environment. In support of this, Harrison et al. [32] previously reported expression of the MYH4 transcript in cultured human fetal primary myotubes, but not in adult human muscle biopsy samples, suggesting that the human MYH4 promoter may only be active during myogenesis. Despite being active during C2C12 myogenesis, the human MYH4 promoter activity remains blunted in comparison to equivalent promoters from MYH4 expressing species, such as pigs (data presented herein) or mice [32]. Taken together, this suggests that the MYH4 promoter sequence plays a critical role in dictating the strength of MYH4 promoter induction during myogenesis and that this disparity in promoter activity can occur independent of nerve innervation in muscle cells. Furthermore, we also show that although both the pig and human MYH4 promoters are responsive to a known MYH4 inducing stimulus, such as MyoD over-expression [10], [30], [32], the absolute capacity to activate the human MYH4 promoter was limited in comparison to the equivalent pig promoter, suggesting that sequence differences between the two promoters dictate the capacity for induction during myogenesis.

We proceeded to elucidate the specific genomic sequence variation responsible for the differential MYH4 promoter activities during myogenesis. Surprisingly, a cluster of just 3 bp mismatches in the proximal CArG and Ebox region (at −74 bp, −68 bp and −48 bp) was responsible for the disparity in pig and human MYH4 promoter activities during myogenesis. The nucleotide mismatch at −74 bp was located in the AT-rich region of a proximal CArG-box (consensus sequence: CC(A/T)_6_GG), resulting in the human CArG-box being a non-consensus CArG-box (CCAAAAAcGG), unlike the equivalent consensus CArG-box in the pig MYH4 promoter (CCAAAAAaGG). This single bp difference was partly responsible for the differential pig and human MYH4 promoter activities during myogenesis. Available literature suggests the CArG-box is a response element that is typically (but probably not exclusively) bound by serum response factor (SRF) and is instrumental for activating many muscle specific genes, particularly genes encoding contractile and structural components of muscle [36], [37]. By deleting the CArG-box and the immediate flanking regions (inclusive of mismatch sites −74 bp and −68 bp) from the MYH4 promoters, we report a critical role of this CArG-box region in regulating the strong transcriptional activation of the porcine MYH4 promoter during myogenesis. Furthermore, by mutating the species-specific mismatches (−74 bp and −68 bp) in the CArG-box region, we confirm that these subtle sequence variations within the CArG-box region are crucial to the species-specific strength of MYH4 promoter induction during myogenesis. This is in agreement with the findings of Harrison et al. [32] who also report that a single bp mutation in the same location (−74 bp) of the AT rich region of the proximal CArG-box is instrumental in dictating the strong induction of the mouse MYH4 promoter. Interestingly, we show that the human non-consensus CArG-box region (inclusive of mismatch sites −74 bp and −68 bp) was still partially functional, as removal of this region was sufficient to moderately reduce promoter activity.

We demonstrated that sequence variation in, and close to, the CArG-box response element (at −74 bp and −68 bp) was of functional significance, with only 1 of the 2 protein-DNA complexes that form with the pig CArG-box region being capable of forming with the human CArG-box region. Sequence variation within CArG-boxes has previously been reported to influence promoter trans-activation by myogenic regulatory factors that bind nearby E-boxes [38] and presents a potential mechanism underlying the differential promoter activities displayed herein.

It is also interesting to speculate whether the close proximity of the 3 bp mismatches identified herein may permit, or restrict, co-operative interactions between factors binding CArG-boxes and E-boxes, since such factors have been shown to physically interact and form trimeric heterodimers [37]. Indeed others have postulated that interactions between CArG and E-boxes may influence Myosin Light Chain 1A gene promoter activation [38]. We show that the removal of a single nucleotide at −68 bp, reducing the gap between the CArG-box and the E-box2, resulted in an increase in promoter activity that, although speculative, may be facilitated by co-operative binding at these two response elements. Therefore, we speculate that species-specific differences in the CArG/E-box region, rather than within a specific response element, are likely crucial to facilitate a strong induction of the porcine MYH4 promoter and that the role of this sequence variation warrants further investigation.

It is important to note that the specific promoter sequence mismatches identified herein between pigs and human are unlikely to account for the lack of MYH4 expression in other non-expressing species (see Figure 5B). For example, the MYH4 promoters of cows and sheep contain a consensus CArG-box sequence (in the equivalent location) yet still display a lack of MYH4 expression. These species do however show mismatches (relative to MYH4 expressing species) within the highly conserved CArG/E-box2 region, supporting our speculation that sequence variation within this region is possibly a critical mechanism underlying differential MYH4 promoter activation. The complexities of protein-DNA interactions between the CArG- and E-box response elements require further investigation to determine their role in regulating the differences in MYH4 gene expression across species.

For the first time, we identify a genomic difference within the MYH4 promoter of pigs and humans that dictate differential promoter activity during myogenesis and postulate that these genomic differences partly explain the different MYH4 expression profiles in these otherwise physiologically similar mammals. We therefore extend the work of Harrison et al. [32] and conclude that the sequence of the CArG-box and its immediate flanking regions, are not only critical for MYH4 expression in small mammals, but is also a region whereby critical sequence variation can permit MYH4 expression in large mammals as well. We also demonstrate that both the pig and human MYH4 promoters are responsive to stimuli (i.e. MyoD) known to induce MYH4 expression, but the capacity for induction is severely influenced by the 3 bp genomic difference identified herein. This work furthers our understanding of genomic mechanisms dictating expression of the fast contracting MYH4 isoform in mammalian skeletal muscle cells.
